# Dietary Silicon Supplementation Improves Egg Production Performance in Late-Phase Laying Hens: Roles of Antioxidant Capacity, Reproductive Hormones, and Serum Cu/Zn Regulation

**DOI:** 10.3390/ani16111731

**Published:** 2026-06-04

**Authors:** Yong Chen, Jiawen Chen, Lei Jin, Shengping Wang

**Affiliations:** 1 College of Animal Science and Veterinary Medicine, Heilongjiang Bayi Agricultural University, Daqing 163319, China; bravech@byau.edu.cn (Y.C.); 15804655664@163.com (J.C.); 2 Hunan Institute of Microbiology, Hunan Academy of Agricultural Sciences, Changsha 410009, China; 13786179543@163.com; 3 Yuelushan Laboratory, Changsha 410009, China

**Keywords:** silica, laying hens, egg laying performance, reproductive hormones, immune indicators, antioxidant capacity, trace elements

## Abstract

Silicon (Si) is an essential trace element involved in multiple physiological processes of animals. This study aimed to investigate the dose-dependent effects of dietary silica (SiO_2_) supplementation on production performance and key blood parameters in laying hens. A total of 360 hens were randomly assigned to five groups (6 replicates/group, 12 hens/replicate) and fed basal diets supplemented with 0% (control), 0.1%, 0.2%, 0.4%, or 0.8% SiO_2_ for 8 weeks. Laying performance, egg quality, serum immune indices, reproductive hormone levels, antioxidant status, and serum trace element concentrations were determined. The results showed that dietary SiO_2_ supplementation significantly affected egg production rate (*p* < 0.05), with the 0.2% group achieving the highest rate compared to the control. For egg quality, yolk weight and yolk thickness were significantly reduced only in the 0.8% group (*p* < 0.05), while other parameters were unaffected (*p* > 0.05). Dietary supplementation with 0.2%, 0.4%, and 0.8% silica significantly increased serum levels of IL-2 and IL-4 (*p* < 0.05), whereas the 0.8% supplementation decreased IL-1 levels (*p* < 0.05). Compared with the control group, serum IgA and IgG levels were elevated in the 0.2%, 0.4%, 0.8% silica-supplemented groups (*p* < 0.05), and serum IgM levels were higher in the 0.4% and 0.8% groups (*p* < 0.05). Regarding reproductive hormones, dietary SiO_2_ significantly increased serum concentrations of β-endorphin, estradiol, growth hormone, luteinizing hormone, and progesterone (*p* < 0.05), with follicle-stimulating hormone elevated in the 0.4% and 0.8% groups (*p* < 0.05). Dietary silica supplementation did not affect serum activities of SOD, GSH-Px, CAT, or T-AOC. Serum POD activity decreased gradually and was significantly lower in the 0.2%, 0.4%, and 0.8% groups than in the control group (*p* < 0.05). Furthermore, SiO_2_ supplementation significantly altered serum Cu and Zn levels (*p* < 0.05), with the 0.8% group having the highest Ca concentration and the 0.1–0.8% groups showing increased Zn levels compared to the control; no effects on Fe and Mn were observed (*p* > 0.05). In conclusion, dietary supplementation with 0.2–0.4% SiO_2_ effectively improves egg production rate, along with enhancing immune function, modulating reproductive hormone secretion, and regulating serum Cu/Zn homeostasis in late-phase laying hens.

## Simple Summary

This study investigated whether adding silicon dioxide (silica), a natural compound, to the diet of laying hens could improve their performance. Over 8 weeks, hens were fed a standard diet or the same diet supplemented with 0.1%, 0.2%, 0.4%, or 0.8% silica. The results showed that hens fed a moderate amount (0.2–0.4% silica) laid the most eggs. This boost in performance was linked to stronger immune systems, beneficial regulation of reproductive hormones, and an improved balance of key minerals like calcium and zinc in the blood. However, adding the highest amount (0.8% silica) decreased the yolk quality of the eggs. These findings suggest that supplementing feed with the optimal amount of silica can be a simple and effective way to support the health and productivity of older laying hens and help to produce eggs more efficiently and sustainably.

## 1. Introduction

Silicon, the second most abundant element in the Earth’s crust, plays a significant role in animal physiology [1]. Research indicates that Si is widely distributed in various tissues, such as bone, skin, hair, nails, and blood vessel walls [2]. It is particularly indispensable for bone formation and mineralization, enhancing bone strength and density by promoting collagen synthesis and regulating calcium–phosphorus metabolism [3]. Dietary silica intake enhances bone health by promoting calcium absorption and deposition, leading to increased bone mineral density and aiding in the prevention of osteoporosis [4]. Silica also benefits intestinal health through its absorptive and regulatory properties: it can bind to harmful pathogens and toxins and modulate the composition of gut microbiota [5,6]. This action helps maintain intestinal microbial homeostasis, improves nutrient digestibility and absorption, and reduces the incidence of intestinal disorders such as constipation [7,8]. Price et al. (2013) demonstrated that silicon is essential for mammalian physiological functions, with silicon deficiency leading to growth retardation, abnormal bone development, and mucosal inflammation [9]. Moreover, exogenous silicon supplementation has been proven to improve skeletal health and enhance tissue repair capacity in animals [10]. The physiological importance of silicon is also verified in human studies, where reduced silicon levels in the aorta are associated with atherosclerosis in the elderly, a condition linked to lipid infiltration in connective tissues and alterations of elastic fibers and the extracellular matrix [11].

Silicon is supplemented in various forms, mainly silicates (e.g., bentonite, zeolite, sodium aluminosilicate) and silicon dioxide (SiO_2_). These silicate-based additives improve poultry production performance through multiple mechanisms, including enhancing intestinal morphological structure, regulating the secretion and activity of digestive enzymes, and adsorbing harmful substances in the gastrointestinal tract. Tauqir et al. (2001) found that dietary supplementation with 2% bentonite significantly improved the growth performance of broilers, with the treatment group showing a 12.3% increase in average daily gain (ADG) and an 8.7% decrease in feed conversion ratio (FCR) compared with the control group [12]. This improvement was attributed to enhanced nutrient utilization, as evidenced by significantly increased ileal digestibility coefficients of dietary energy and protein. Santurio et al. (1999) demonstrated that dietary silicates exert a dual modulatory effect on the gut microbiota: inhibiting the proliferation of pathogenic bacteria (e.g., *Escherichia coli*) and promoting the colonization of beneficial taxa (e.g., *Bifidobacterium* and *Lactobacillus*) to restore intestinal microbial homeostasis and enhance nutrient absorption [13]. In layer production, silicon supplementation has multiple beneficial effects on egg production and egg quality. Beyond improving laying production parameters (including laying rate and egg weight), it significantly enhances the ultrastructure of eggshells and reduces egg breakage rates [13]. The beneficial effect of silicon on poultry bone health is likely attributed to its specific crystalline properties. Sodium zeolite, with its unique three-dimensional porous structure, not only facilitates a sustained release of silicate ions but also adsorbs harmful gases like ammonia in poultry houses, thus contributing to an improved rearing environment [5]. In laying hens, dietary silicon supplementation significantly enhances the expression of calcium-binding proteins in the eggshell gland, which promotes calcium deposition in eggshells, reduces the incidence of eggshell defects, and extends the laying cycle. Furthermore, silicon may optimize eggshell quality by regulating the secretion of parathyroid hormone and the activation of vitamin D, thereby synergistically maintaining calcium–phosphorus homeostasis in laying hens [14].

Faryadi and Sheikhahmadi (2017) reported that dietary supplementation with 500–4000 mg/kg nanosilica in 16- to 26-week-old laying quails significantly increased egg weight and egg quality and significant elevations in bone ash, calcium, and silicon contents were observed [3]. The effects of combined supplementation with 200 ppm activated silica and 0.12% betaine on the production performance of quails demonstrated that the combined regimen significantly improved growth performance during the starter phase and effectively increased both laying rate and egg weight during the early laying period [15]. Samy and Elsherif (2025) reported that nanosilica can increase the growth performance of broilers by enhancing their antioxidant capacity and improving bone health [4]. Skeletal integrity and mineral homeostasis are critical for laying hens, especially in the late laying phase: to meet the high metabolic demand for calcium and phosphorus during intense egg production, late-phase laying hens often suffer from mineral homeostasis imbalance, which can lead to an increased incidence of osteoporosis and impaired eggshell integrity [16].

Studies have shown that in the late laying period of hens, increased oxidative stress, imbalanced calcium metabolism, impaired immune function, and reduced hormone secretion contribute to ovarian dysfunction and decreased bone quality. These factors lead to declines in laying performance and egg quality in late-laying hens [17,18,19]. This study systematically evaluated the dose-dependent effects of dietary silicon dioxide supplementation on serum biochemical indices, antioxidant status, reproductive hormone profiles, and serum calcium/zinc homeostasis in late-phase laying hens. We hypothesize that dietary silica supplementation improves egg quality and production performance by enhancing blood physiological responses, health status, and regulating reproductive functions.

## 2. Materials and Methods

### 2.1. Experimental Animals, Diets, and Feeding Management

A total of 360 fifty-week-old Lohmann laying hens with comparable initial egg production rates and body weights were randomly allocated into five experimental groups using a completely randomized design, with six replicates per group and twelve hens per replicate. The control group (0%) received a basal diet throughout the trial period, while the treatment groups were fed the basal diet supplemented with 0.1%, 0.2%, 0.4% and 0.8% food-grade silica (purity 99%; Tianjin Longhua Chengxin Powder Technology Co., Ltd., Tianjin, China) on top. The experiment lasted for 8 weeks and was terminated at 57 weeks of age. Laying hens were housed in three-layer, stair-step cages equipped with automatic drinkers and egg collectors (each cage measured 350 mm × 350 mm × 450 mm × 400 mm (width × depth × front height × rear height), with a cage floor slope of 6°). The hens had a 16 h light time per day (supplemented with artificial lighting in the morning and evening). The poultry house was routinely supervised. Hens were fed twice daily at 08:00 and 17:00 and had free access to water by nipple drinkers. Manure was removed regularly. Eggs were collected twice daily at 10:00 and 16:00 and labeled by group. Hens received essential vaccinations including Marek’s disease (1 d), ND + IB combined vaccine (7 d), and IBD vaccine (14 d), followed by routine boosters during the rearing period. Flock disinfection was performed routinely every 5 days. The composition and nutrient content of the basal diets are shown in Table 1. This study was approved by the Animal Care and Use Committee on Animal Ethics of Heilongjiang Bayi Agricultural University (DWKJXY94).

The premix provides the following nutrients per kilogram of diet: VA 10,000 IU, VD 3000 IU, VE 15 IU, VB1 3 mg, VB_6_ 6 mg, VB_2_ 8 mg, VB_12_ 50 mg, nicotinic acid 50 mg, folic acid 1.5 mg, copper 12.5 mg, iron 90 mg, zinc 82.8 mg, manganese 95.4 mg, and iodine 0.19 mg. The nutritional levels are calculated values.

### 2.2. Determination of Laying Performance and Egg Quality Parameters

The total number of eggs and individual egg weights were recorded per treatment group (*n* = 6). The egg laying rate and average egg weight were calculated as follows:

Egg laying rate (%) = (Number of eggs/Number of hens) × 100

Average egg weight (g) = Total egg weight/Number of eggs laid

Daily feed intake and leftover feed were recorded each day to calculate the average daily feed intake and feed conversion ratio (FCR).

At the end of the experiment (57 weeks of age), five eggs were randomly selected from each replicate to analyze the egg quality parameters (*n* = 30), including Haugh unit, egg weight, albumen height, eggshell strength, yolk color, and eggshell thickness, were measured by the Integrated Egg Quality Tester System (ETU-01C; Huayi Saifu Technology Co., Ltd., Shanghai, China). For eggshell thickness, the shell membrane was removed, and measurements were taken at the blunt end, equatorial region, and sharp end; the average of these three values was recorded. Eggshell and yolk weights were measured using an electronic balance (Dr. Mega CX-12000, Yancheng Yunlian Intelligent Technology Co., Ltd., Yancheng, China).

### 2.3. Serum Sample Collection and Analyses of Immune Index, Oxidant Levels, Serum Hormone, and Trace Element

At the end of the experiment (57 weeks of age), two hens were randomly selected from each replicate to collect blood samples from the wing vein (*n* = 12). The samples were centrifuged at 3000 r/min for 15 min to obtain serum, which was then stored at −20 °C.

Serum concentrations of immunoglobulin A (IgA), immunoglobulin M (IgM), immunoglobulin G (IgG), interleukin-1β (IL-1β), interleukin-2 (IL-2), interleukin-4 (IL-4), and vitellogenin were determined using ELISA assay kits (Nanjing Jianjian Bioengineering Research Institute, Nanjing, China) and strictly following the instructions.

Serum superoxide dismutase (SOD), glutathione peroxidase (GSH-Px), peroxidase (POD), catalase (CAT), total antioxidant capacity (T-AOC) activities, and malondialdehyde (MDA) content were measured using ELISA kits (Nanjing Jiancheng Bioengineering Institute, Nanjing, China), according to the instructions.

Serum concentrations of luteinizing hormone (LH), progesterone (P4), β-endorphin (EP), estradiol (E2), growth hormone (GH), and follicle-stimulating hormone (FSH) were measured using ELISA kits (Nanjing Jiancheng Bioengineering Institute, Nanjing, China), according to the instructions.

The concentrations of iron (Fe), calcium (Ca), magnesium (Mg), zinc (Zn), and copper (Cu) in blood were determined by ELISA kits (Nanjing Jiancheng Bioengineering Institute, Nanjing, China), according to the instructions.

### 2.4. Statistical Analysis

The experimental data were analyzed using one-way analysis of variance (ANOVA) in SPSS software (version 25.0; IBM Corp., Armonk, NY, USA), and Duncan’s test for multiple comparisons, with *p* < 0.05 as the criterion for determining the significance of differences. The results are presented as mean ± standard error.

## 3. Results

### 3.1. Effects of Dietary Silica Supplementation on Laying Performance

The laying rate of Lohmann hens presented a trend of increasing initially and then decreasing as dietary silica supplementation increased (Table 2). The 0.2% silica group exhibited the highest egg production rate in the 1–4 weeks, the 5–8 weeks, and the entire experimental period. This increase was statistically significant compared to the control group for the 1–4 weeks and the overall period (*p* < 0.05). No significant difference in laying rate was observed between the 0.2% and 0.4% silica groups (*p* > 0.05). The addition of silica to the diet of laying hens did not affect the average daily feed intake and feed conversion ratio.

### 3.2. Effects of Dietary Silica Supplementation on Egg Quality of Laying Hens

Dietary silica supplementation significantly affected egg yolk weight and thickness (Table 3). Compared to the 0.4% silica group, the yolk weight of eggs produced by the 0.8% group was significantly lower (*p* < 0.05). Yolk weight in the 0.4% group, however, did not differ significantly from that in the control or other treatment groups (*p* > 0.05). Similarly, yolk thickness was significantly reduced only in the 0.8% group compared to all others (*p* < 0.05), with no significant differences observed among the remaining groups (*p* > 0.05). No other egg quality parameters were significantly influenced by silica supplementation (*p* > 0.05).

### 3.3. Effects of Dietary Silica Supplementation on Serum Immune Indices

**Figure 1:**
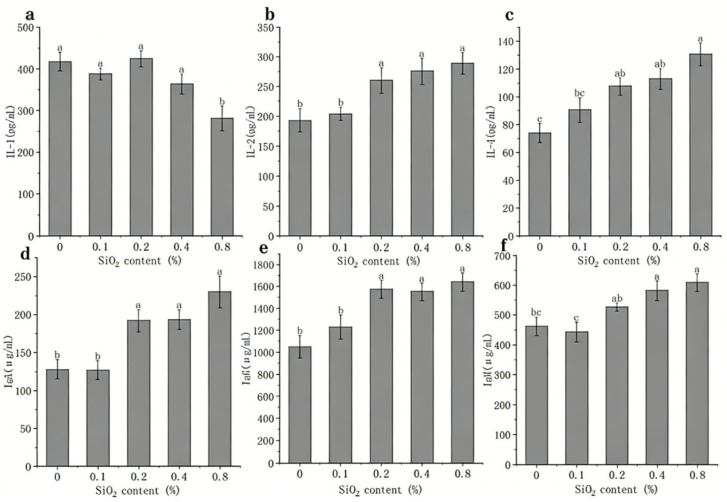
Effects of different silica additions to diets on immunity indexes of laying hens. Different lowercase letters on bars mean significant differences at *p* < 0.05 for each subfigure.

The effects of dietary silica addition on the immune parameters of laying hens are shown in Figure 1. Serum IL-1 levels were significantly reduced only in the 0.8% silica group compared to the control (*p* < 0.05; Figure 1a), with no significant changes observed in other treatment groups. In contrast, serum concentrations of IL-2 and IL-4 increased progressively with silica dosage and were significantly elevated in the 0.2%, 0.4%, and 0.8% groups compared to the control (*p* < 0.05; Figure 1b,c).

Compared with the control group, serum levels of IgA and IgG were significantly higher in the 0.2%, 0.4%, and 0.8% groups (*p* < 0.05; Figure 1d,e). Serum IgM levels were significantly elevated in the 0.4% and 0.8% groups compared with both the control and 0.1% group (*p* < 0.05; Figure 1f).

### 3.4. Effects of Dietary Silica Supplementation on Serum Hormones

**Figure 2:**
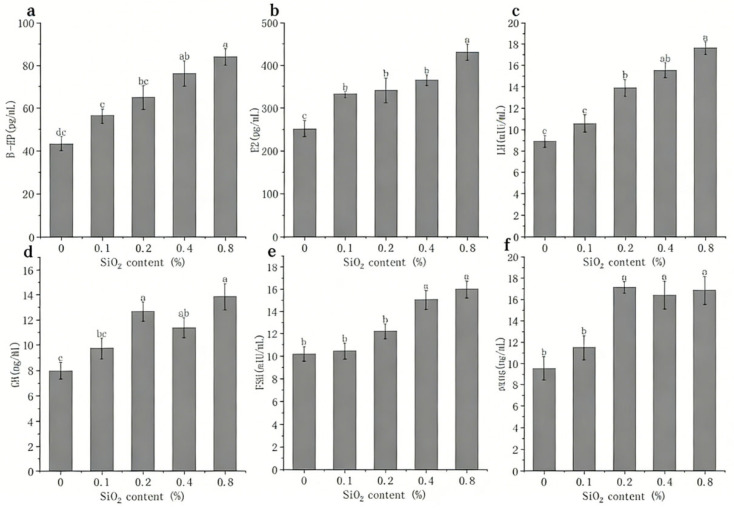
Effect of different silica additions in diets on blood hormone levels in laying hens. Different lowercase letters on bars mean significant differences at *p* < 0.05 for each subfigure.

Serum hormone levels of laying hens showed a dose-dependent increase with the increase in dietary silica supplementation (Figure 2). Compared to the control group, silica addition significantly elevated serum concentrations of both β-endorphin (Figure 2a) and estradiol (Figure 2b) (*p* < 0.05). Serum levels of growth hormone, luteinizing hormone and progesterone levels were significantly higher in the 0.2%, 0.4% and 0.8% silica groups (*p* < 0.05; Figure 2c,d,f), and serum follicle-stimulating hormone (FSH) levels were significantly elevated in the 0.4% and 0.8% silica groups (*p* < 0.05; Figure 2e).

### 3.5. Effects of Dietary Silica Supplementation on Antioxidant Status

The effects of dietary silica on blood antioxidant indexes are shown in Table 4. The activities of serum superoxide dismutase (SOD), glutathione peroxidase (GSH-Px), catalase (CAT) and total antioxidant capacity (T-AOC) were unaffected by dietary silica supplementation. Serum peroxidase (POD) activity exhibited a gradual decreasing trend, and the 0.2%, 0.4%, and 0.8% groups had significantly lower POD activity than the control group (*p* < 0.05). Serum malondialdehyde (MDA) content exhibited a trend of initially decreasing and then increasing response to increasing silica levels. MDA contents was lowest in the 0.1% silica group and significantly lower than in the control group, 0.4% and 0.8% groups (*p* < 0.05).

### 3.6. Effects of Dietary Silica Supplementation on Serum Mineral Elements

The effects of dietary silica supplementation on blood mineral elements are shown in Table 5. Dietary silica supplementation significantly altered serum copper (Cu) concentrations (*p* < 0.05). The highest Cu level was observed in the 0.1% silica group, which was markedly higher than that in the control, 0.4%, and 0.8% groups (*p* < 0.05). Serum zinc (Zn) concentrations were significantly elevated in the 0.1%, 0.2%, and 0.8% silica-supplemented groups relative to the control group (*p* < 0.05). Dietary silica supplementation exerted no significant effects on serum iron (Fe), calcium (Ca), or magnesium (Mg) concentrations (*p* > 0.05).

## 4. Discussion

Silicon plays a beneficial role in bone health by enhancing calcium absorption and metabolism, thereby improving bone stability. It also aids in maintaining intestinal homeostasis and preventing intestinal disorders through its adsorptive properties. Incharoen et al. (2021) reported that silicon supplementation could improve bone strength and laying rate with no adverse effects on egg quality [20]. The diet of broiler chickens being supplemented with nanosilica improves growth performance, and Samy and Elsherif (2025) pointed out that this may be attributed to enhanced antioxidant capacity and improved bone health [4]. Tran et al. (2015) demonstrated that dietary 0.02% SiO_2_ supplementation significantly increased body weight in turkeys [21]. A report on chicken showed that adding 100 mg/kg silica to feed increased the growth rate [22]. As reviewed by Burton et al. (2025), bioavailable silica plays a crucial role as an essential ultra-trace mineral in poultry growth and development, with silicon-containing compounds having been shown to affect live weight and feed efficiency [23].When the diet of quails was supplemented with 4000 mg/kg nanosilica, it significantly increased egg weight and egg quality in quails [3]. However, nanosilica supplementation had no significant effects on feed intake, egg production, or feed conversion ratio (FCR) in laying quails. In the present study, dietary silica supplementation (0.2%) improved the egg production performance of laying hens. The 0.4% silica group exhibited the best egg quality among all treatment levels. Dietary SiO_2_ supplementation did not affect feed intake or FCR in laying hens, similar to previous observations in quails. Our dose-dependent trend suggests that a moderate silicon (0.4%) optimizes reproductive metabolism, while excess (0.8%) disrupts yolk quality (reduced yolk weight/thickness).

In terms of immune indicators, this study found that, except for IL-1, the content of IL-2, IL-4, IgA, IgG, and IgM in laying hens showed an upward trend in response to increasing dietary silica. This indicates that an appropriate dose of silica (0.2–0.4%) can effectively enhance the humoral and cellular immune function of laying hens. The observed reduction in IL-1 may be explained by silica’s potential to inhibit specific signal transduction pathways, thereby reducing the release of pro-inflammatory cytokines [24]. However, a high supplementation level (0.8%) of silica may induce immune cell stress and suppress immune function [25]. A study on 16-week-old calves fed a compound feed additive containing organic selenium, acidifiers, and nano-silicon dioxide showed no effects on serum concentrations of interferon-γ (IFN-γ), interleukin-1β (IL-1β), and tumor necrosis factor-α (TNF-α). Serum immunoglobulin (Ig), interleukin-2 (IL-2), interleukin-4 (IL-4), and interleukin-10 (IL-10) exhibited a tendency to increase uniformly, indicating a beneficial effect on calves [26]. Ojha et al. (2025) reported that the immune responses of 5- to 7-day-old crossbred calves were not affected by dietary silicon supplementation [27]. The differences between this calf study and the present study may be attributed to inconsistent selection of indicators for humoral and cell-mediated immunity or to species differences (laying hens vs. calves).

Endorphins, as key neurotransmitters in regulating stress and behavior, play a crucial role in animal welfare and production performance. Ortega et al. (2022) reported that appropriate supplementation of trace minerals stimulated the neuroendocrine system and promoted endorphin secretion, thereby effectively alleviating stress responses and enhancing pig welfare [28]. In the present study, compared to the control group, serum endorphin levels increased significantly with dietary silica supplementation (from 0.1% to 0.8% of silica). This led to an increase in the egg production rate, especially at supplementation levels of 0.2% and 0.4%.

Estradiol plays a central role in avian reproductive physiology and is critically involved in key processes such as follicular development and ovulation. A study conducted on women aged 50 to 62 years revealed a significant association between dietary silicon intake and hip bone mineral density in those receiving hormone replacement therapy and confirmed that estradiol is essential for the normal physiological function of silicon in bone [11]. Boron is involved in steroidogenesis and vitamin metabolism, regulating the synthesis of sex hormones (e.g., testosterone and estradiol) and promoting the activation of vitamin D. Melenikioti et al. (2026) proposed that silicon is similarly implicated in these processes, for example, by modulating parathyroid hormone and vitamin D activity to support collagen synthesis and maintain skeletal homeostasis [29]. In this study, serum estradiol levels increased gradually with dietary silica supplementation, reaching a peak in the 0.8% group. Some studies have shown that mineral elements and their compounds can modulate endocrine homeostasis in birds, influencing estrogen synthesis and secretion and thereby affecting reproductive performance. Elnesr et al. (2024) found that supplementing laying hen diets with trace mineral complexes significantly increased serum estradiol levels and improved egg-laying performance [30]. The observed elevation of estradiol by silica in our study similarly suggests its potential to positively modulate reproductive function in hens. Luteinizing hormone (LH) and follicle-stimulating hormone (FSH) are key regulators of the avian reproductive cycle. In this experiment, both LH and FSH levels increased with higher dietary silica. Qin et al. (2026) reported that mineral additives can influence gonadotropin secretion [31]. These results suggest that silica may enhance follicular development and ovulation in laying hens by regulating gonadotropin levels, indicating its significance in optimizing reproductive performance. Progesterone plays an indispensable role in maintaining pregnancy and regulating the reproductive cycle. In this study, progesterone levels were significantly elevated in the 0.2%, 0.4%, and 0.8% silica-supplemented groups compared to the control group. This finding is consistent with the report by Pereira et al. (2020) that mineral elements can influence progesterone secretion [32]. These results suggest that dietary silica may help stabilize progesterone levels in laying hens, potentially benefiting their reproductive health.

Growth hormone (GH) plays a crucial role in regulating animal growth, development, and metabolism. In the present study, dietary silica supplementation was positively correlated with increased serum GH levels in laying hens. This observation is consistent with findings by Muszyński et al. (2018) in broilers, where certain additives were shown to stimulate GH secretion and enhance growth performance [33]. Supplementation of the diet with silicon significantly increased serum GH levels in Japanese quail [34]. Studies on broilers have shown that dietary silicon (zeolite) supplementation also significantly elevated growth hormone levels [35].

Oxidative stress impairs the normal physiological function of shell gland cells, suppresses the expression and synthesis of calcium-binding proteins, and inhibits calcium transport. However, the antioxidant effects of silicon reported in previous studies remain inconsistent. For instance, dietary nanosilica supplementation improved growth performance in broilers by enhancing antioxidant capacity [4]. Silicon also exerts beneficial antioxidant effects that help maintain laying performance in late-phase laying hens [36]. Plant-derived silicon supplementation was found to enhance bone strength and laying rate [20]. In contrast, other studies reported negative effects: Ahamed (2013) observed a dose-dependent reduction in SOD activity in A549 cells exposed to nanosilica [37], and Fan et al. (2014) demonstrated that nanosilica decreased intracellular GSH-Px levels and induced oxidative damage in human bronchial epithelial cells [38]. The present study showed that serum POD activity declined with increasing dietary silica levels. Such discrepancies may be attributed to differences in supplementation dosage, animal species, and silicon source. These factors deserve further investigation in future research.

Minerals including Fe, Ca, Mn, Zn, and Cu serve as indispensable cofactors or coenzymes for antioxidant enzymes and play a critical role in maintaining enzyme activity and redox balance [39]. Adequate intake of trace elements can alleviate oxidative stress, enhance reproductive performance, and facilitate the repair of oxidative DNA damage [40]. In the present study, dietary silica supplementation significantly increased serum Zn concentrations in all treated groups compared with the control, indicating that silica effectively improves Zn status in late-phase laying hens. Serum Cu content was markedly elevated in the 0.1% silica group but declined progressively with further increasing silica levels. This dose-dependent regulation of Cu homeostasis likely reflects a self-adjusting physiological mechanism, which merits in-depth exploration in future research. Pritchard et al. (2020) reported that silicon supplementation in one-day-old broilers decreased serum boron levels and increased serum calcium concentrations but had no effects on bone mineral density, morphology or mechanical strength [41]. In a study on 6-week-old female Wistar rats fed diets with graded concentrations of calcium and silicon, Kim and Choi (2021) found that silicon supplementation significantly elevated blood osteocalcin levels, whereas a high-calcium diet significantly reduced them [42]. Bone mineral density increased significantly with higher dietary calcium intake. Furthermore, silicon supplementation notably enhanced tibial strength in rats fed with adequate dietary calcium, suggesting that the synergistic interaction between calcium and silicon is dose-dependent. Some studies have demonstrated that silicon has a minor impact on the contents of mineral elements (e.g., Si, Ca, and B) in bone, and it primarily participates in collagen synthesis to improve bone density and strength [11]. Melenikioti et al. (2026) proposed that silicon’s regulatory role in hormonal metabolism may be indirect, and it acts more by enhancing systemic integrity through synergistic participation in the metabolic pathways of other metallic elements [29]. Higher utilization of mineral elements and enhanced immune function may reduce oxygen free radicals and peroxides in the body. This could explain the observed decrease in antioxidant enzyme levels while improving laying performance.

## 5. Conclusions

Dietary supplementation with 0.2–0.4% silicon dioxide effectively improves the laying performance of late-phase laying hens, as evidenced by the significantly elevated egg production rate. These beneficial effects are associated with enhanced immune function, optimized secretion of reproductive hormones, and regulated serum Zn and Cu levels. Although SiO_2_ supplementation decreased POD activity, the supplementation range of 0.2–0.4% had positive effects on most indicators, while excessive addition (0.8%) may adversely affect yolk quality.

## Figures and Tables

**Table 1:** Ingredient Composition and Nutritional Levels of the Basal Diet for Laying Hens.

Diet Composition	Proportion/%	Nutritional Level	Content/%
Corn	62.00	Crude Protein	17.00
Soybean Meal	24.00	Metabolizable Energy/(MJ/Kg)	11.44
Soybean Oil	1.00	Calcium	3.57
Fish Meal	2.00	Total Phosphorus	0.54
Dicalcium Phosphate	1.00	Available Phosphorus	0.34
Salt (NaCl)	0.32	Methionine	0.41
Limestone	9.00	Lysine	0.90
Threonine	0.10		
Methionine	0.12		
Choline Chloride	0.10		
Premix	0.30		
Phytase	0.02		
Lysine	0.04		
Total	100.00		

**Table 2:** Effect of different dietary silica additions on egg-laying performance of Lohmann hens.

Items		0%	0.1%	0.2%	0.4%	0.8%	*p*
Laying rate (%)	1–4 week	94.27 ± 0.68 ^b^	94.06 ± 0.46 ^b^	97.60 ± 0.56 ^a^	97.29 ± 0.37 ^a^	93.02 ± 0.75 ^b^	0.007
	5–8 week	87.50 ± 0.83	90.21 ± 1.13	90.31 ± 0.91	88.75 ± 1.45	88.54 ± 1.18	0.352
	1–8 week	90.71 ± 0.62 ^b^	91.98 ± 0.61 ^ab^	93.95 ± 0.67 ^a^	92.78 ± 0.91 ^ab^	90.54 ± 0.79 ^b^	0.010
Average daily feed intake (g)	1–4 week	119.42 ± 1.12	118.63 ± 1.88	122.49 ± 3.27	117.95 ± 1.01	116.34 ± 1.31	0.256
	5–8 week	117.23 ± 1.01	116.51 ± 1.70	119.57 ± 2.90	115.70 ± 0.97	115.18 ± 1.27	0.432
	1–8 week	118.32 ± 1.07	117.58 ± 1.82	120.82 ± 3.23	116.79 ± 0.93	115.75 ± 1.49	0.416
Feed conversion ratio	1–4 week	2.26 ± 0.05	2.14 ± 0.07	2.18 ± 0.05	2.21 ± 0.05	2.20 ± 0.06	0.635
	5–8 week	2.23 ± 0.05	2.13 ± 0.05	2.17 ± 0.04	2.19 ± 0.05	2.14 ± 0.03	0.625
	1–8 week	2.24 ± 0.06	2.13 ± 0.05	2.17 ± 0.07	2.19 ± 0.04	2.16 ± 0.05	0.457

Different lowercase letters in same row mean significant differences at *p* < 0.05.

**Table 3:** Effect of different silica additions to diets on egg quality of laying hens.

Items	0%	0.1%	0.2%	0.4%	0.8%	*p*
Egg weight (g)	59.16 ± 0.76	59.10 ± 0.50	59.07 ± 0.61	59.65 ± 0.72	57.77 ± 0.63	0.330
Egg yolk weight (g)	15.29 ± 0.31 ^ab^	15.16 ± 0.21 ^ab^	15.26 ± 0.21 ^ab^	15.61 ± 0.24 ^a^	14.73 ± 0.25 ^b^	0.036
Eggshell weight (g)	5.34 ± 0.14	5.24 ± 0.07	5.33 ± 0.09	5.32 ± 0.10	5.27 ± 0.08	0.906
Egg white weight (g)	37.93 ± 0.47	38.08 ± 0.43	37.90 ± 0.44	38.10 ± 0.54	37.01 ± 0.45	0.986
Harris unit	92.80 ± 1.37	92.35 ± 1.19	91.65 ± 1.43	91.15 ± 1.27	88.95 ± 1.08	0.082
Egg yolk thickness (mm)	18.31 ± 0.22 ^a^	18.09 ± 0.19 ^a^	18.07 ± 0.19 ^a^	18.16 ± 0.17 ^a^	17.50 ± 0.14 ^b^	0.028
Egg white thickness (mm)	8.72 ± 0.27	8.63 ± 0.24	8.47 ± 0.26	8.57 ± 0.93	7.87 ± 0.19	0.989
Eggshell thickness (mm)	0.28 ± 0.01	0.29 ± 0.01	0.31 ± 0.01	0.29 ± 0.01	0.31 ± 0.01	0.104
Maximum effort (N)	0.80 ± 0.18	0.78 ± 0.18	0.85 ± 0.19	0.99 ± 0.22	0.64 ± 0.14	0.773
Deformation of the force point (mm)	1.50 ± 0.09	1.51 ± 0.06	1.60 ± 0.08	1.59 ± 0.09	1.43 ± 0.07	0.765
Eggshell strength (N)	2.68 ± 0.12	2.78 ± 0.11	2.87 ± 0.12	2.77 ± 0.14	2.78 ± 0.09	0.767
Eggshell color	11.88 ± 0.24	12.10 ± 0.32	11.18 ± 0.35	11.58 ± 0.34	11.60 ± 0.33	0.195

Different lowercase letters in same row mean significant differences at *p* < 0.05.

**Table 4:** Effects of different silica additions in diets on blood antioxidant indexes in laying hens.

Items	0%	0.1%	0.2%	0.4%	0.8%	*p*
SOD (U/mL)	587.79 ± 23.08	512.22 ± 18.66	565.87 ± 10.23	546.61 ± 21.97	551.68 ± 13.46	0.078
GSH-PX (U/mL)	1019.96 ± 40.24	956.21 ± 50.05	960.67 ± 46.23	1072.62 ± 56.71	1144.57 ± 51.81	0.086
POD (U/mL)	11.56 ± 1.01 ^a^	6.11 ± 1.13 ^bc^	7.47 ± 1.02 ^b^	4.44 ± 0.51 ^c^	5.22 ± 0.90 ^bc^	0.000
CAT (U/mL)	0.85 ± 0.03	0.61 ± 0.06	0.83 ± 0.09	0.63 ± 0.14	0.81 ± 0.27	0.051
T-AOC (mmol/L)	0.66 ± 0.04	0.63 ± 0.08	0.60 ± 0.06	0.63 ± 0.05	0.55 ± 0.05	0.701
MDA (nmol/mL)	4.16 ± 0.23 ^ab^	2.85 ± 0.27 ^c^	3.53 ± 0.28 ^bc^	4.19 ± 0.47 ^ab^	4.82 ± 0.39 ^a^	0.007

Different lowercase letters in same row mean significant differences at *p* < 0.05.

**Table 5:** Effects of different silica additions in diets on serum elements in laying hens.

Items	0%	0.1%	0.2%	0.4%	0.8%	*p*
Fe (mg/L)	10.34 ± 0.73	10.76 ± 0.48	11.48 ± 0.34	11.75 ± 0.60	10.55 ± 0.59	0.181
Cu (μmol/L)	7.28 ± 0.76 ^b^	15.05 ± 2.58 ^a^	10.94 ± 1.76 ^ab^	6.97 ± 0.87 ^b^	6.84 ± 0.65 ^b^	0.030
Mg (mmol/L)	0.62 ± 0.05	0.66 ± 0.03	0.70 ± 0.04	0.71 ± 0.05	0.76 ± 0.05	0.362
Zn (μmol/L)	34.11 ± 3.76 ^b^	46.95 ± 2.70 ^a^	46.04 ± 0.50 ^a^	41.40 ± 4.02 ^ab^	44.28 ± 5.18 ^a^	0.044
Ca (mmol/L)	2.59 ± 0.11	2.35 ± 0.13	2.71 ± 0.15	2.72 ± 0.18	2.97 ± 0.15	0.059

Different lowercase letters in same row mean significant differences at *p* < 0.05.

## Data Availability

The data are available on request.
